# Supplementary material for: Network based multifactorial modelling of miRNA-target interactions
Source: PeerJ. 2021 Mar 19;9:e11121. doi: 10.7717/peerj.11121 (PMC7983860; doi:10.7717/peerj.11121)
Supplement: Supplemental Information 1 [file peerj-09-11121-s001.pdf]

Supplementary Figures and Tables

Selcen Ari Alper Yilmaz

2021-02-19

Contents

2. Supplementary Figures **2**

2.1 Sample data set analysis in absence of interaction factors. . . . 3

2.2 Calculations with interaction factors 5

2.3 Sample+ data set analysis with interaction factors. . . . 6

2.4 Common target perturbation in Sample+ data set. . . . 8

2.5 Runtime Analysis 11

3 Supplementary Tables **11**

3.1 *Sample+* data set 11

3.2 Significant factors in miRNA:target interactions 12

3.3 Content of High-throughput experimental studies 13

3.4 Variables of network object during simulation 13

3.5 Comparison of perturbation efficiency between Sample and Sample+ data set 14

4. Notes and Access to code **14**

REFERENCES **15**

2. Supplementary Figures

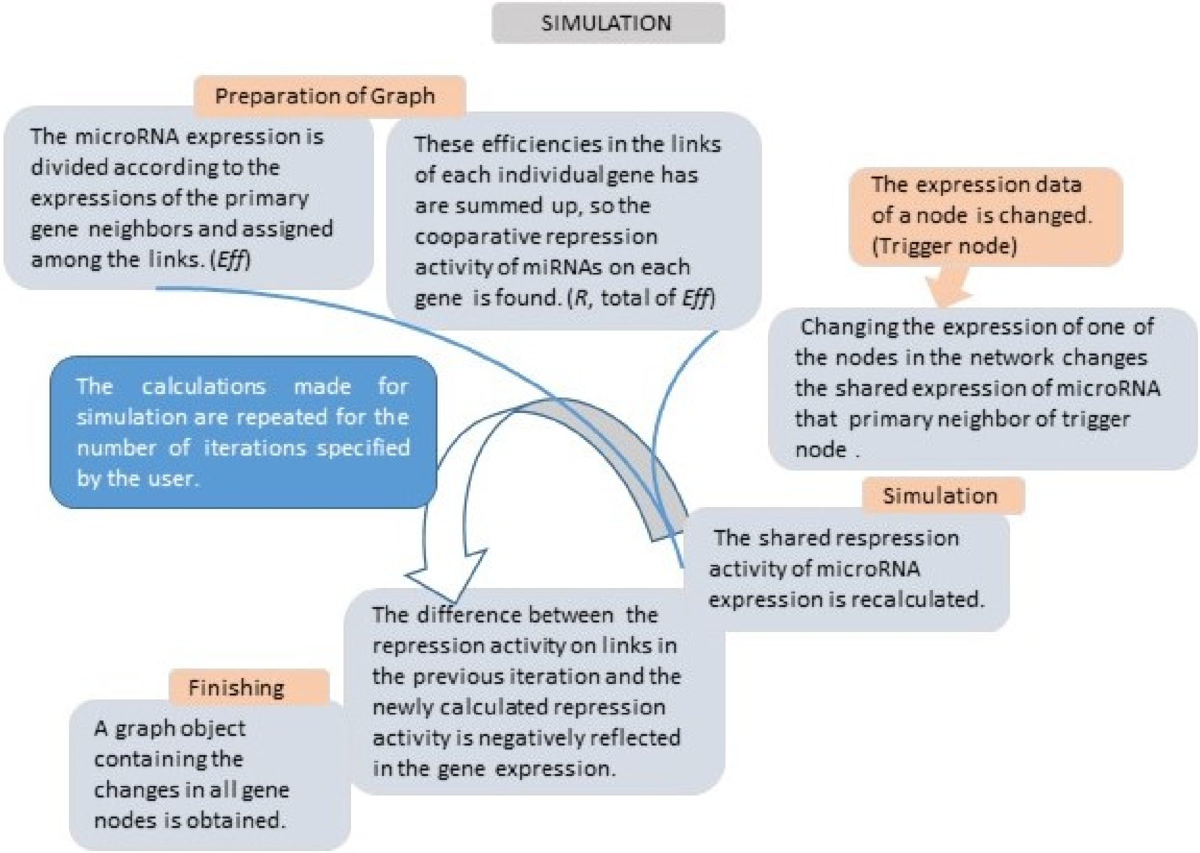

Figure S1: Workflow for simulation of competing endogenous RNA regulations. Graph object in steps is saved and updated continuously in simulation.

2.1 Sample data set analysis in absence of interaction factors.

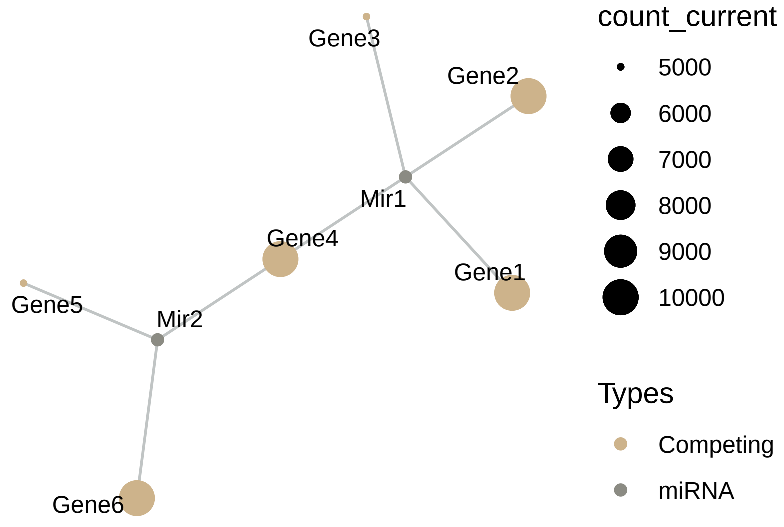

Figure S2: Sample Data set in Steady-state. Inıtial expression levels in minsamp network (*Sample* network in manuscript). The network contains two miRNAs and 6 genes with arbitrarily chosen expression values. Refer to Table S1 for exact expression values

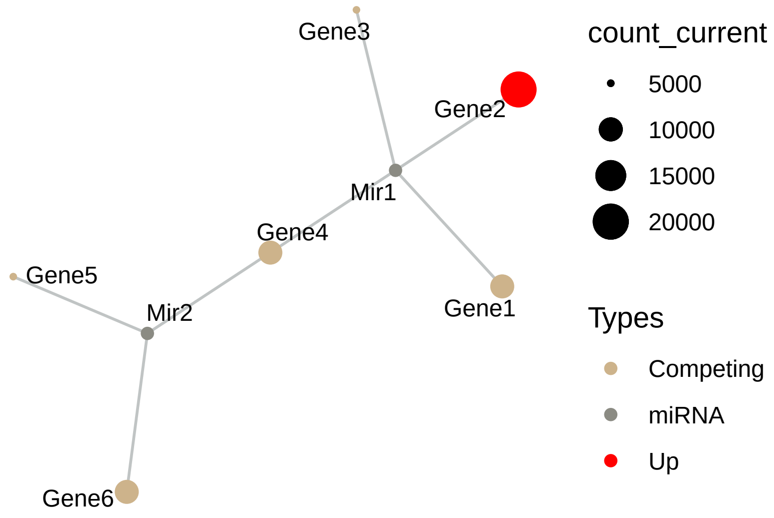

Figure S3: Gene2 Upregulation on Sample Data set. Expression level of Gene2 is increased from 10,000 to 20,000 in order to demonstrate the calculation steps.

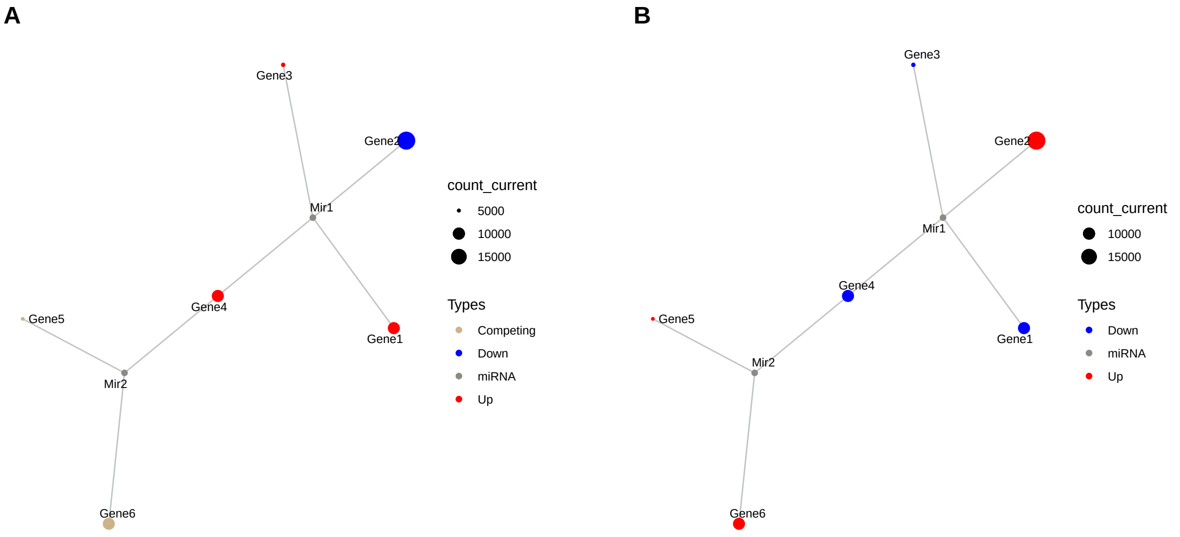

Figure S4: Sequential iteration of Sample data after up-regulation of Gene2. A) First response of network to Gene2 upregulation (2nd iteration). B) Spreading of perturbation on system (3th iteration)

2.2 Calculations with interaction factors

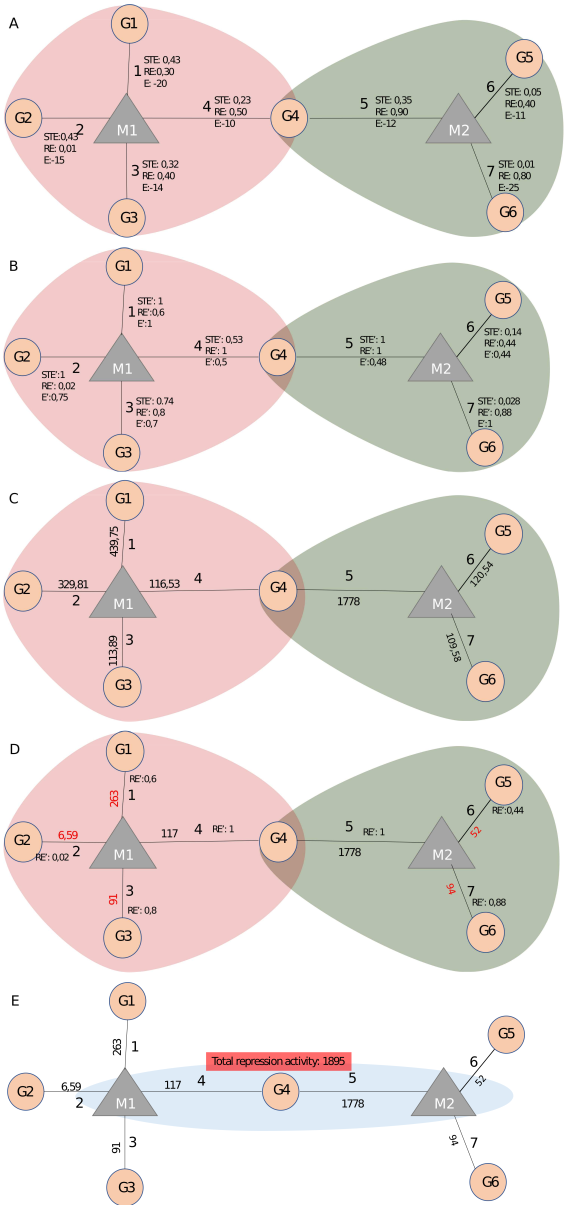

Figure S5: Calculation of initial miRNA repression level (counts) using interaction parameters in Sample+ network. A) Interaction paramaters between genes and miRNAs in Sample+ are shown on network while expression levels can be found in Table S1. B) Interaction paramaters were updated after normalisation C) Amount of miRNA distributed to each mRNA according to mRNA levels and affinity parameters (Energy and Seed Type Effect) are shown on edges. D) Values on edges indicate degredation level (couns). Red values indicate degredation level affected by region effect (RE) parameter. E) Total repression on G4 from two miRNAs is calculated by summing repression values originating from both miRNAs.

G, Gene; M, miRNA; STE, seed type effect; RE, Region Effect; E, Energy; STE', normalized values of seed type effect; RE', normalized values of region effect parameter; E', normalized values of Energy parameter. Numbering on edges match the pair order in Table S1.

2.3 Sample+ data set analysis with interaction factors.

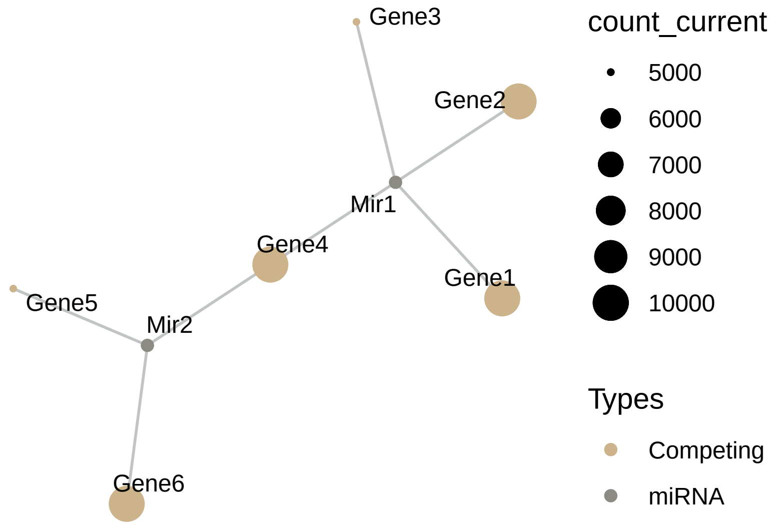

Figure S6: Sample+ in Steady-state. Interaction factors of Sample+ network are available in Table S1.

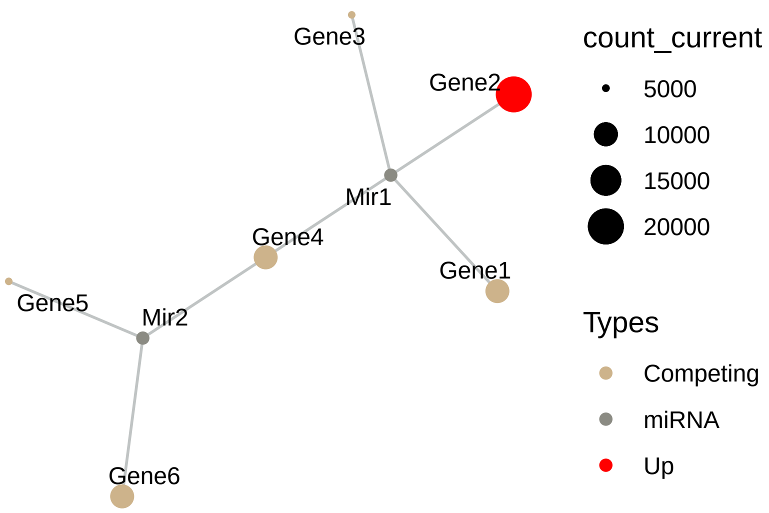

Figure S7: Perturbation in Sample+ network by two-fold increase in Gene2 expression level.

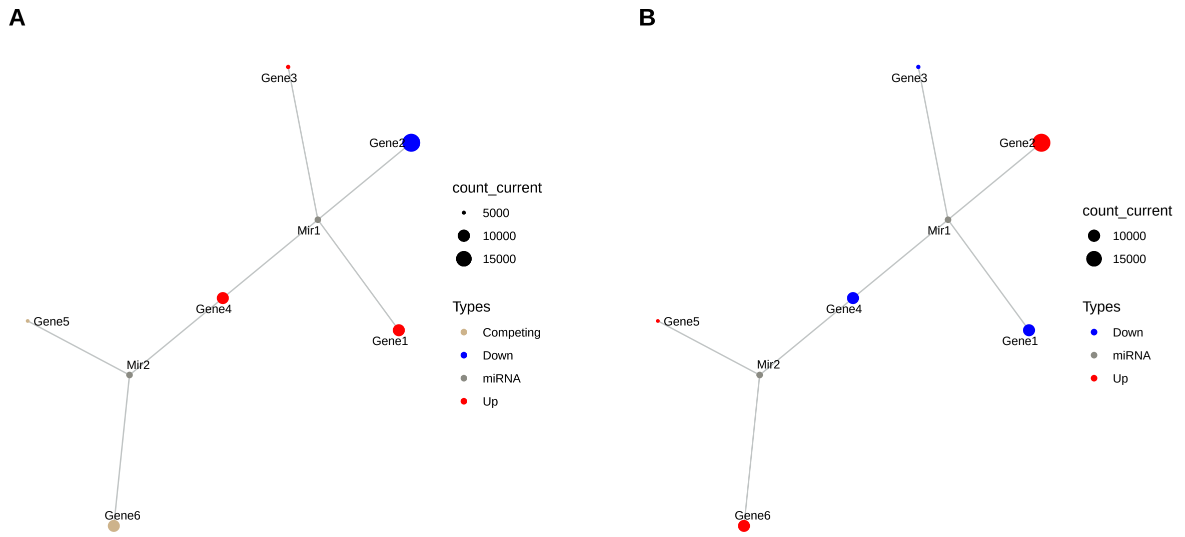

Figure S8: Sequential iteration of Sample+ A) First response of network to Gene2 upregulation (2nd iteration). B) Spreading of perturbation on system (3rd iteration). Although visualisation looks similar to Figure S4B, current counts of genes are drastically different.

2.4 Common target perturbation in Sample+ data set.

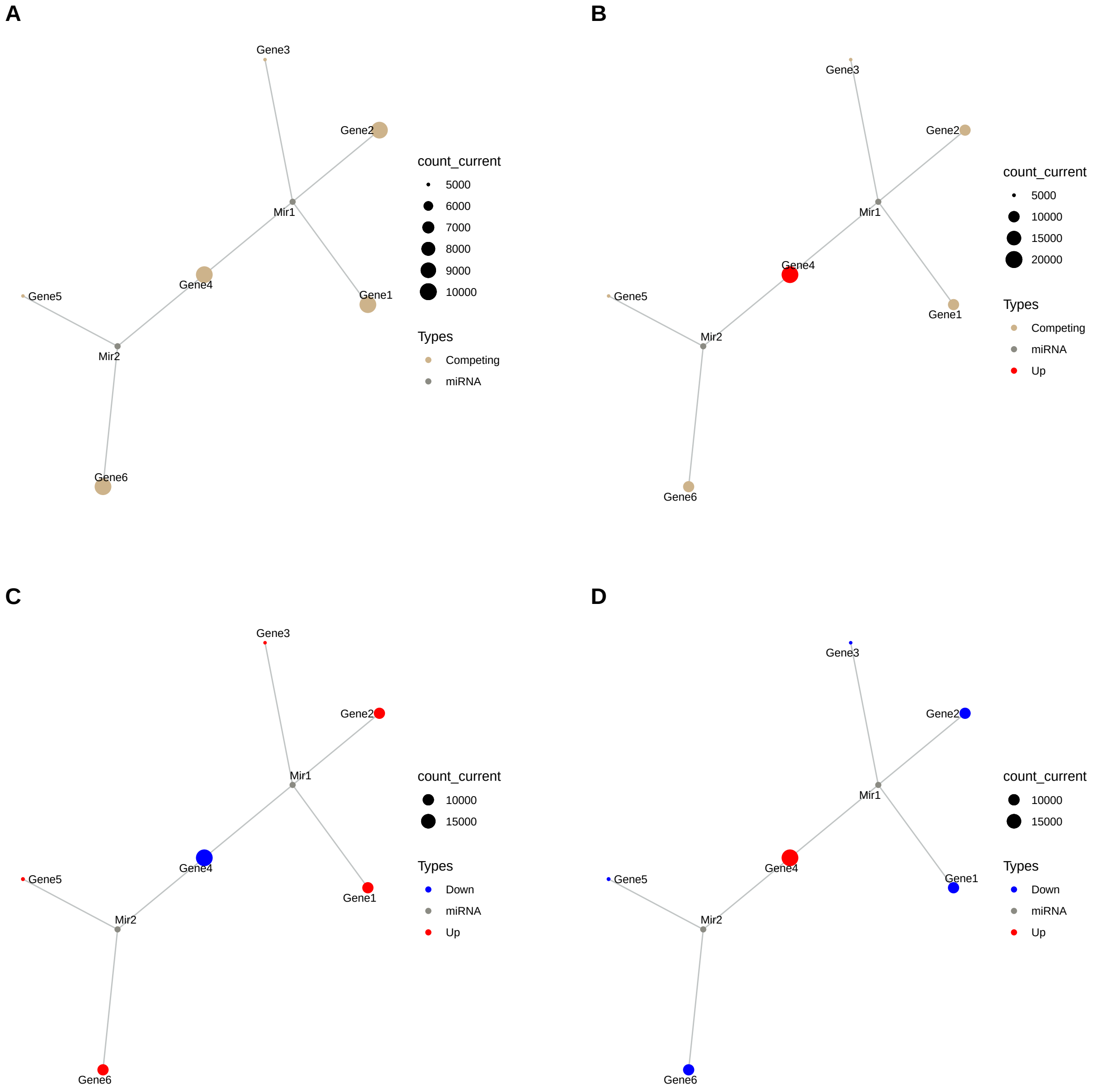

Figure S9: Perturbation of Gene4 and its effects on Sample+. A) Network at steady-state. B) Upregulation of Gene4. C) Primary response of network to upregulation of Gene4. D) Re-regulation of whole nodes on system (3th iteration)

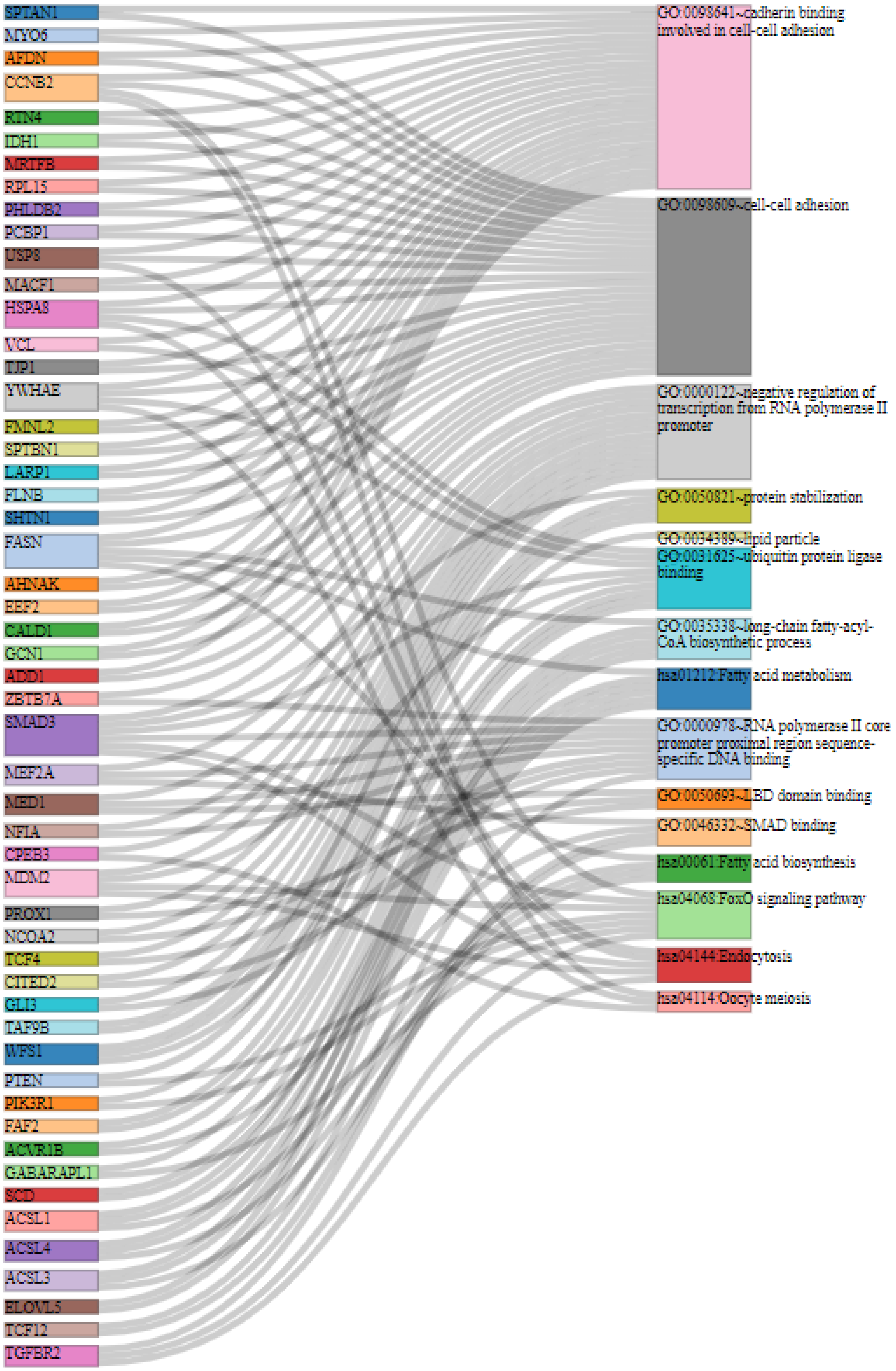

Figure S10: Sankey diagram represents top five KEGG and GO (molecular function and biological process) terms and genes enriched on these terms. Genes with single edge are not shown.

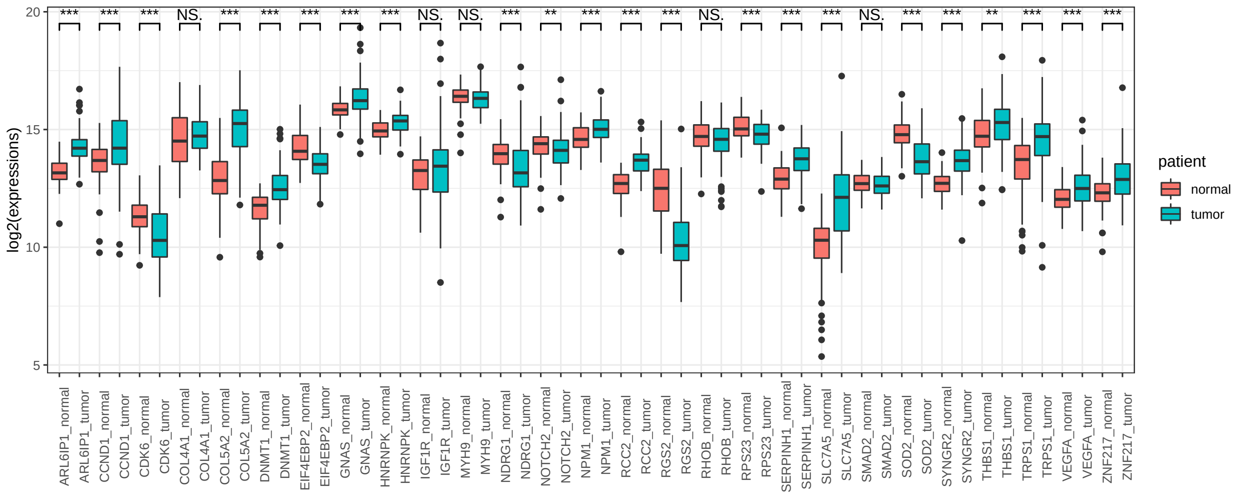

Figure S11: Log2 transformed expression levels of tumor-specific perturbing nodes in tumor and normal tissue samples of 87 patients for Real network.

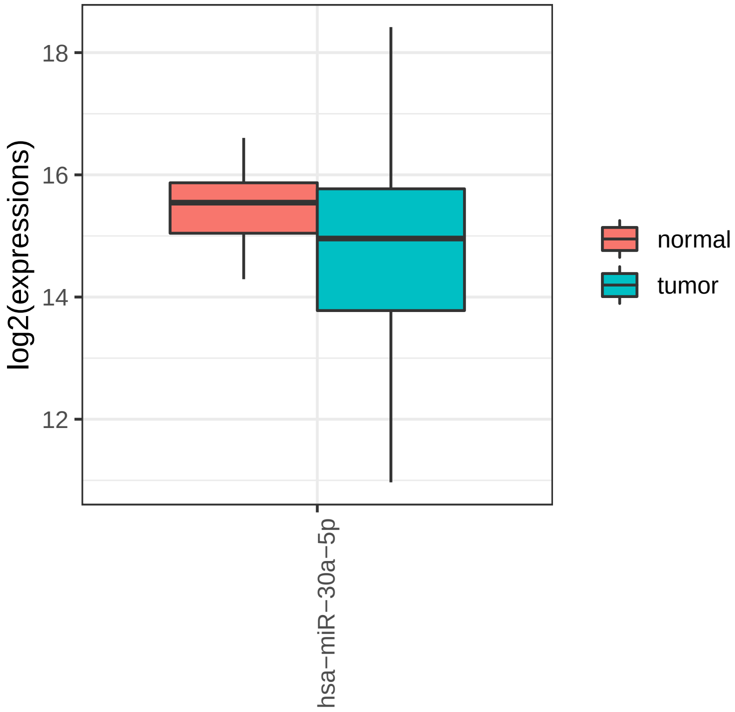

Figure S12: Log2 transformed expression level of miR-30a-5p in tumor and normal tissue samples of 87 patients for Real data set.

2.5 Runtime Analysis

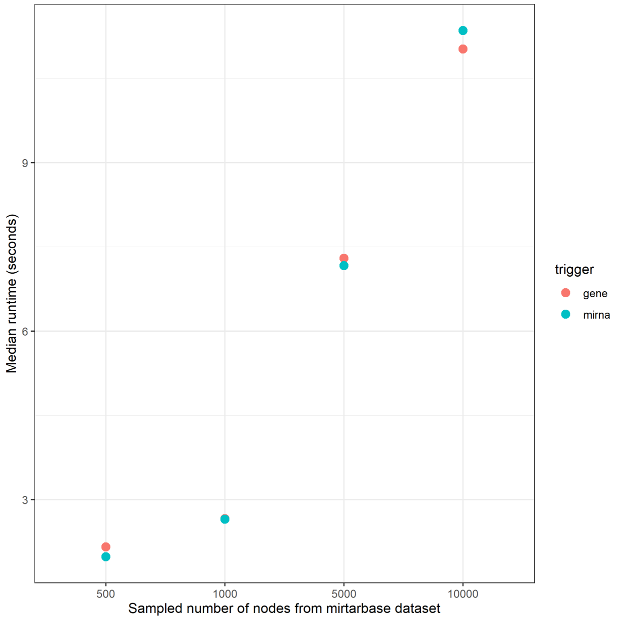

Figure S13: Simulation runtime comparison of sampled networks with size 500, 1000, 5000 and 10000

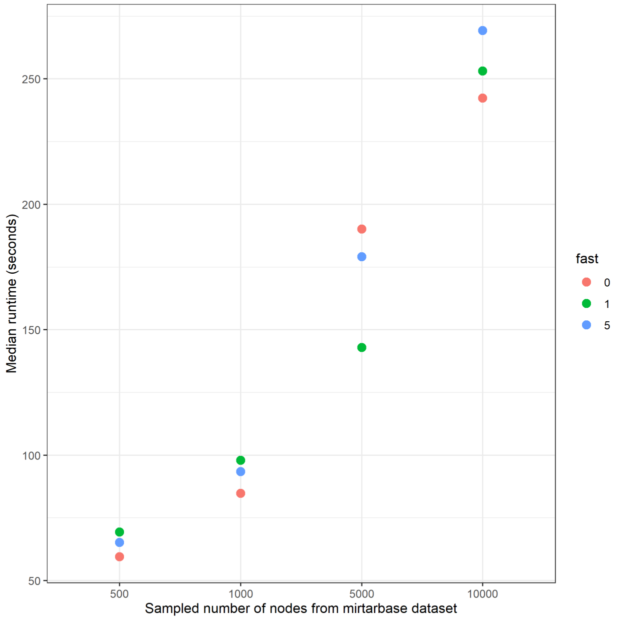

Figure S14: Perturbation efficiency evaluation function runtime comparison of sampled networks with size 500, 1000, 5000 and 10000

3 Supplementary Tables

3.1 *Sample+* data set

Table S1: The parameters which affect miRNA:target interactions (i.e. seed type, region, energy) are provided in Sample+ data set, while these factors are not utilized in simulation of Sample data set.

Competing	miRNA	Competing Expression	miRNA Expression	Seed Type Effect	Region Effect	Energy
Gene1	Mir1	10000	1000	0.43	0.30	-20
Gene2	Mir1	10000	1000	0.43	0.01	-15
Gene3	Mir1	5000	1000	0.32	0.40	-14
Gene4	Mir1	10000	1000	0.23	0.50	-10
Gene4	Mir2	10000	2000	0.35	0.90	-12
Gene5	Mir2	5000	2000	0.05	0.40	-11
Gene6	Mir2	10000	2000	0.01	0.80	-25

3.2 Significant factors in miRNA:target interactions

Some of information about miRNA:target interactions were exhibited directly by high-throughput studies. On the other hand, we were examined other interaction parameters based on different studies.

- (Helwak et al. 2013; Moore et al. 2015) reported the energy values in miRNA:target interactions.
- Comparisons of canonical seed types were evaluated by study of (Grimson et al. 2007), while functional and non-functional seed interactions were studied by (Bartel 2009) and (Betel et al. 2010).
- Numeric definition of target region location effect was performed based on studies of (Hausser et al. 2013) and (Helwak et al. 2013).

Table S2: Efficiency factors for seed types.

seed type	seed type effect
6-mer_noncanonical	0.05
9-mer	0.43
6-mer	0.07
8-mer	0.43
7-mer	0.23
none	0.01
5-mer_noncanonical	0.04
5-mer	0.05
6-merA1_noncanonical	0.05
7-mer-8m_noncanonical	0.21
7-mer-8m	0.25
8-mer_noncanonical	0.35
7-merA1_noncanonical	0.16
7-merA1	0.19
6-merA1	0.07

Table S3: Efficiency factors for binding regions on targets

region	region effect
3UTR	0.84
CDS	0.42

region	region effect
3UTRCDS	0.93
5UTR	0.01
5UTRCDS	0.42
none	0.01
intron	0.01
CDS3UTR	0.93
CDS5UTR	0.42
exon_unclassified	0.20
CDS3UTRintron	0.93
3UTRintron	0.84
CDSintron	0.42
5UTRintron	0.01
5UTR3UTR	0.93
CDS5UTR3UTR	0.93

3.3 Content of High-throughput experimental studies

Table S4: Context of miRNA:target pairs supported by High-throughput Experiments. CLEAR-CLiP and CLASH data sets were integrated as described in Section 2 of Supplementary Material and Method.

Variable	Definition
cluster	Barcode from experimental method
chromosome	Chromosome of Target gene from raw data
start_position	Gene start position from raw data
end_position	Gene end position from raw data
strand	Gene strand
hgnc_symbol	Gene name (Symbol)
Ensembl_Gene_Id	Ensembl Gene Id of gene
Ensembl_Transcript_Id	Ensembl transcript id of mRNA of Target gene
target_seq	mRNA sequences targeted by miRNA
miRNA	miRNA id (from miRBase version 21)
miR_seq	miRNA sequence
seed_type	seed type of miRNA:target interaction
Energy	Energy of miRNA:target binding
HG38build_loc	Recent chromosomal location of Gene
Genome_build	Genome build of given chromosome, start and end positions
region	interaction location on target
region_effect	Coefficient of location on target
seed_type_effect	Coefficient for seed sequence of miRNA:target interaction

3.4 Variables of network object during simulation

As a result of simulation a data set, a graph object is obtained that includes various variables in edge and node data. A graph object includes variables at Table S5.

Table S5: The context graph object during the process.

Variables	Description
Node Variables	
name	node name
type	Competing or miRNA
node_id	in on graph object
initial_count	Initial Expression value of node
count_pre	Expression value of node at previous regulation
count_current	Existing expression value of node
changes_variable	Regulation of node (Up, down or steady)
Edge Variables	
Competing name	name of genes
miRNA name	name of miRNAs
Competing expression	Expression values of competing elements at steady-state
miRNA expression	Expression values of miRNA elements at steady-state
energy	coefficient of miRNA:target interactions (binding affinity)
seed type	coefficient of miRNA:target interactions (binding affinity)
region	coefficient of miRNA:target interactions (degradation efficiency)
afff factor	coefficient scaled and combined affinity factor
degg factor	coefficient scaled and combined degradation factor
comp_count_list	list of competing expression for each iteration
comp_count	pre: competing expression at previous iteration; current: competing expression at present iteration
mirna_count_list	list of miRNA expression for each iteration
mirna_count	pre: miRNA expression at previous iteration; current: miRNA expression at present iteration
effect	pre: total miRNA reppressive effect on individual target at previous iteration ; current: miRNA reppressive effect on individual target at present iteration
effect_list	list of miRNA reppressive effect on individual target for each iteration

3.5 Comparison of perturbation efficiency between Sample and Sample+ data set

Table S6: Perturbation efficiencies of nodes in Sample+ and Sample data set. PE, perturbation efficiency; PC, perturbed node count.

Data set		Sample+		Sample
**Name**	**PE**	**PC**	**PE**	**PC**
Gene1	0.132	2	0.268	3
Gene2	0.198	3	0.268	3
Gene3	0.0555	2	0.150	3
Gene4	0.197	4	0.870	5
Gene5	0.143	1	0.358	2
Gene6	0.131	1	0.619	2
Mir1	0.806	3	1.638	4
Mir2	2.80	3	3.432	3

4. Notes and Access to code

- *Table S7*: refers functional annotations of all highly perturbing genes from simulations of network retrieved from miRTarBase.
- *Table S8*: refers functional annotations of tumor specific highly perturbing genes achieved from simulations of network retrieved from miRTarBase.
- *Table S9*: refers functional annotations of tumor specific highly perturbing genes achieved from simulations of network retrieved from miRNA:target pairs that are validated by high-throughput experimental studies.

REFERENCES

Bartel, David P. 2009. “MicroRNAs: Target Recognition and Regulatory Functions.” *Cell* 136 (2): 215–33. https://doi.org/10.1016/j.cell.2009.01.002.

Betel, Doron, Anjali Koppal, Phaedra Agius, Chris Sander, and Christina Leslie. 2010. “Comprehensive Modeling of microRNA Targets Predicts Functional Non-Conserved and Non-Canonical Sites.” *Genome Biology* 11 (8): R90.

Grimson, Andrew, Kyle Kai-How Farh, Wendy K. Johnston, Philip Garrett-Engele, Lee P. Lim, and David P. Bartel. 2007. “MicroRNA Targeting Specificity in Mammals: Determinants Beyond Seed Pairing.” *Molecular Cell* 27 (1): 91–105. https://doi.org/10.1016/j.molcel.2007.06.017.

Hausser, J., A. P. Syed, B. Bilen, and M. Zavolan. 2013. “Analysis of CDS-Located miRNA Target Sites Suggests That They Can Effectively Inhibit Translation.” *Genome Research* 23 (4): 604–15. https://doi.org/10.1101/gr.139758.112.

Helwak, Aleksandra, Grzegorz Kudla, Tatiana Dudnakova, and David Tollervey. 2013. “Mapping the Human miRNA Interactome by CLASH Reveals Frequent Noncanonical Binding.” *Cell* 153 (3): 654–65. https://doi.org/10.1016/j.cell.2013.03.043.

Moore, Michael J., Troels K. H. Scheel, Joseph M. Luna, Christopher Y. Park, John J. Fak, Eiko Nishiuchi, Charles M. Rice, and Robert B. Darnell. 2015. “miRNA-Target Chimeras Reveal miRNA 3’-End Pairing as a Major Determinant of Argonaute Target Specificity.” *Nature Communications* 6 (November): 8864. https://doi.org/10.1038/ncomms9864.
