# Supplementary material for: Network based multifactorial modelling of miRNA-target interactions
Source: PeerJ. 2021 Mar 19;9:e11121. doi: 10.7717/peerj.11121 (PMC7983860; doi:10.7717/peerj.11121)
Supplement: Supplemental Information 2 [file peerj-09-11121-s002.pdf]

Supplementary Materials And Methods

Selcen Ari Alper Yilmaz

2021-02-19

Contents

1. Determination of perturbation efficiencies of nodes in network. 1
1.1 Sample Data set: 2
1.2 Sample+ Data set: 2

2. Additional data manipulation steps 3
2.1 Arrangement of CLASH data set 3
Query of Human Genome 19. 3
Adding miRNA and gene information 3
Converting CLASH data to human genome 38 build. 4
Interpreting the CLASH seed structures in data set 5
2.2 Arrangement of CLEAR-CLiP data set (Moore et al. 2015) 5
Query of Human Genome 18 6
Adding Genome Information to data set 6
Converting human genome build 6
Seed type manipulation in CLEAR-CLiP data set 8
2.3 Integration of two experimental data set 8
2.4 Adding Coefficients of Interaction factors 8

3. Functions defined for ceRNA models and workflow of method 10

4. Perturbation analysis of Real/Real+ networks 10

REFERENCES 10

The functions that are used in the this study are developed with R and are available in Bioconductor.

1. Determination of perturbation efficiencies of nodes in network.

`find_node_perturbation` function runs `calc_perturbation` function for each node with given parameters. In this particular example up-regulation parameter was two, iteration parameter was three. By using formulas described in manuscript perturbation efficiency and number of affected nodes (perturbed count) were calculated. Results for *Sample* network are shown below.

1.1 Sample Data set:

```
library(ceRNAnetsim)
data("minsamp")  # refers Sample and Sample+ data


sample_graph <- priming_graph(minsamp, competing_count = Competing_expression,
    miRNA_count = miRNA_expression)

find_node_perturbation(sample_graph, how = 2, cycle = 3,
    limit = 0.1) %>% as_tibble() %>% select(name, perturbation_efficiency,
    perturbed_count)
```

```
## # A tibble: 8 x 3
##   name  perturbation_efficiency perturbed_count
##   <chr>                   <dbl>           <dbl>
## 1 Gene1                   0.268               3
## 2 Gene2                   0.268               3
## 3 Gene3                   0.150               3
## 4 Gene4                   0.871               5
## 5 Gene5                   0.358               2
## 6 Gene6                   0.619               2
## 7 Mir1                    1.64                4
## 8 Mir2                    3.43                3
```

1.2 Sample+ Data set:

Results for *Sample+* network are shown below.

```
sample_graph <- priming_graph(minsamp, competing_count = Competing_expression,
    miRNA_count = miRNA_expression, aff_factor = c(energy,
        seed_type), deg_factor = region)

find_node_perturbation(sample_graph, how = 2, cycle = 3,
    limit = 0.1) %>% as_tibble() %>% select(name, perturbation_efficiency,
    perturbed_count)
```

```
## # A tibble: 8 x 3
##   name  perturbation_efficiency perturbed_count
##   <chr>                   <dbl>           <dbl>
## 1 Gene1                  0.132                2
## 2 Gene2                  0.198                3
## 3 Gene3                  0.0555               2
## 4 Gene4                  0.197                4
## 5 Gene5                  0.143                1
## 6 Gene6                  0.131                1
## 7 Mir1                   0.806                3
## 8 Mir2                   2.80                 3
```

2. Additional data manipulation steps

2.1 Arrangement of CLASH data set

CLASH data set was retrieved from Helwak et. al (Helwak et al. 2013).

```
clash_url <- "https://www.ncbi.nlm.nih.gov/pmc/articles/PMC3650559/bin/mmc1.txt"

clashelwak <- read_tsv(clash_url, comment = "#", skip = 1)

# hg19
```

Query of Human Genome 19.

Human genome 19 information was handled through biomaRt package.

```
library(biomaRt)  #can be installed via BiocManager

# listMarts(host = 'http://grch37.ensembl.org')
# #HG19
ensemblgrch37 <- useMart(host = "http://grch37.ensembl.org",
    biomart = "ENSEMBL_MART_ENSEMBL", dataset = "hsapiens_gene_ensembl")

hg19 <- getBM(attributes = c("ensembl_transcript_id",
    "ensembl_gene_id", "chromosome_name", "start_position",
    "end_position", "hgnc_symbol", "entrezgene_id",
    "strand"), mart = ensemblgrch37)
```

Adding miRNA and gene information

```
clashelwak <- clashelwak %>% separate(microRNA_name,
    c("Barcode", "Database", "mirna_name", "type"),
    sep = "_") %>% separate(mRNA_name, c("Ensembl_Gene_Id",
    "Ensembl_Transcript_Id", "Hugo_Symbol", "mRNA_Type"),
    sep = "_") %>% rename_with(~str_replace(., "'",
    "_"), contains("UTR"))
```

miRNA releases were obtained from miRBase. In this step, release 21 (in Human genome 38) was downloaded.

```
mirbase_url <- "ftp://mirbase.org/pub/mirbase/21/genomes/hsa.gff3"

mirbasehg38 <- read_tsv(mirbase_url, comment = "#",
    col_names = FALSE) %>% filter(X3 != "miRNA_primary_transcript") %>%
    separate(X9, c("ID", "Alias", "Name", "Precusor"),
        sep = ";") %>% mutate(ID = substr(ID, 4, length(ID)),
    Alias = substr(Alias, 7, length(Alias)), Name = substr(Name,
        6, length(Name)), Precusor = substr(Precusor,
        14, length(Precusor))) %>% dplyr::select(chr = X1,
    start = X4, end = X5, strand = X7, ID, Alias, Name,
    Precusor)
```

Since CLASH data set was published according to Human Genome 19 version, we used miRBase release 15.

```
mirbase15_url <- "ftp://mirbase.org/pub/mirbase/15/database_files/mirna_mature.txt.gz"

clashelwakfinal <- read_tsv(mirbase15_url, col_names = FALSE) %>%
    filter(str_starts(X2, "hsa")) %>% dplyr::select(mirna_ID = X2,
    mirbase_ID = X3) %>% inner_join(mirbasehg38 %>%
    dplyr::select(ID, Name), by = c(mirbase_ID = "ID")) %>%
    dplyr::select(mirbase_ID, Name) %>% distinct() %>%
    inner_join(clashelwak, by = c(mirbase_ID = "Barcode")) %>%
    dplyr::select(Name, miRNA_seq, Ensembl_Gene_Id,
        Ensembl_Transcript_Id, Hugo_Symbol, mRNA_seq_extended,
        chimeras_decompressed, seed_type, seed_basepairs,
        folding_class, seq_ID, folding_energy, `5_UTR`,
        CDS, `3_UTR`) %>% inner_join(hg19, by = c(Ensembl_Gene_Id = "ensembl_gene_id",
    Ensembl_Transcript_Id = "ensembl_transcript_id",
    Hugo_Symbol = "hgnc_symbol")) %>% mutate(region1 = ifelse(`5_UTR` ==
    "1", "5UTR", " "), region2 = ifelse(`3_UTR` ==
    "1", "3UTR", " "), region3 = ifelse(CDS == "1",
    "CDS", " ")) %>% unite(region, c(region1, region2,
    region3), sep = "||") %>% dplyr::select(chromosome_name,
    start_position, end_position, strand, Hugo_Symbol,
    Ensembl_Gene_Id, Ensembl_Transcript_Id, mRNA_seq_extended,
    Name, miRNA_seq, seq_ID, seed_type, seed_basepairs,
    folding_class, folding_energy, region)
```

Converting CLASH data to human genome 38 build.

There are different liftover methods for conversion among Human Genome builds. We preferred to use UCSC liftover tool

```
# Obtaining chromosomal locations from miRNA:target
# interaction data set.

clashelwakfinal %>% dplyr::select(1, 2, 3) %>% unite(start_end,
    c("start_position", "end_position"), sep = "-") %>%
    mutate(Chromosome = paste0("chr", chromosome_name,
        "")) %>% unite(chromosome_name, c("Chromosome",
    "start_end"), sep = ":") %>% write_tsv("lift19.txt",
    col_names = FALSE)

# In liftover site, Original Assembly was chosen as
# hg19 and New Assembly was chosen as hg38. After
# we uploaded 'lift19.txt' file to UCSC Liftover
# site, the output 'View Conversions' was
# downloaded as hg38clashcomp (new locations of the
# genes on HG38), 'Display failure file' was
# download as 'lift19_del.txt' (deleted regions on
# the HG38 genome build)

lift19_del <- read_tsv("data/lift19_del.txt", col_names = FALSE,
    comment = "#") %>% rename(chromosome_loc = X1) %>%
    dplyr::filter(str_starts(chromosome_loc, "chr")) %>%
```

```
    separate(chromosome_loc, c("Chr", "End"), "-",
        remove = TRUE) %>% separate(Chr, c("Chr", "Start"),
    ":", remove = TRUE) %>% mutate(Start = as.numeric(Start),
    End = as.numeric(End))

# removing deleted location from CLASH data set

clashelwakfinal <- clashelwakfinal %>% mutate(Chromosome = paste0("chr",
    chromosome_name, "")) %>% dplyr::anti_join(lift19_del,
    by = c(Chromosome = "Chr", start_position = "Start",
        end_position = "End"))
# adding new location information:

clashelwakfinal <- clashelwakfinal %>% bind_cols(read_tsv("data/hg38clashcomp.txt",
    col_names = FALSE))


# Arrangement in data set

clashelwakfinal <- clashelwakfinal %>% rename(HG38build_loc = X1) %>%
    mutate(Genom_build = "hg19") %>% dplyr::select(cluster = seq_ID,
    chromosome = Chromosome, start_position, end_position,
    strand, hgnc_symbol = Hugo_Symbol, Ensembl_Gene_Id,
    Ensembl_Transcript_Id, target_seq = mRNA_seq_extended,
    miRNA = Name, miR_seq = miRNA_seq, seed_type, seed_type2 = seed_basepairs,
    seed_type3 = folding_class, Energy = folding_energy,
    HG38build_loc, Genom_build, region) %>% mutate(strand = as.character(strand))
```

Interpreting the CLASH seed structures in data set

```
clashelwakfinal <- clashelwakfinal %>% mutate(seed_type = ifelse(seed_type ==
    "noncanonical_seed" & seed_type2 > 4 & seed_type3 ==
    "I", paste0(seed_type2, "-mer"), seed_type), seed_type = ifelse(seed_type ==
    "noncanonical_seed" & seed_type2 > 4 & seed_type3 ==
    "II", paste0(seed_type2, "-mer_noncanonical"),
    seed_type), seed_type = ifelse(seed_type == "noncanonical_seed" &
    seed_type2 > 4 & seed_type3 == "III", paste0(seed_type2,
    "-mer_noncanonical"), seed_type), seed_type = ifelse(seed_type ==
    "noncanonical_seed" & seed_type2 > 4 & seed_type3 ==
    "IV", paste0(seed_type2, "-mer_noncanonical"),
    seed_type), seed_type = ifelse(startsWith(seed_type,
    "no"), "none", seed_type)) %>% dplyr::select(-seed_type2,
    -seed_type3)
```

2.2 Arrangement of CLEAR-CLiP data set (Moore et al. 2015)

CLEAR-CLiP data set was retrieved from Moore et.al. Please note that we need Supplementary Data 4, however there was error in website, you need to click on Supplementary Data 3 to download the data.

```r
clear_clip_url <- "https://static-content.springer.com/esm/art%3A10.1038%2Fncomms9864/MediaObjects/4146[illegible]


download.file(url = clear_clip_url, destfile = "CLEAR-CLIP.xlsx",
    mode = "wb")

clearclip <- readxl::read_excel("CLEAR-CLIP.xlsx",
    1, skip = 23)

# Clearclip hg18
```

Query of Human Genome 18

```r
# HG18
library(biomaRt)

# listMarts(host = 'may2009.archive.ensembl.org')
ensembl54 = useEnsembl(host = "may2009.archive.ensembl.org",
    biomart = "ensembl", dataset = "hsapiens_gene_ensembl",
    mirror = "asia")


hg18 <- getBM(attributes = c("ensembl_transcript_id",
    "ensembl_gene_id", "chromosome_name", "start_position",
    "end_position", "hgnc_symbol", "entrezgene_id",
    "strand"), mart = ensembl54)

# should there be any connection problems, read the
# pre-saved version of hg18 gene information by
# commenting out the following line

# hg18 <- read_tsv('data/hg18.txt') %>%
# rename_with(~ tolower(.x) %>% str_replace_all('
# ','_') %>% str_remove('_\\(bp\\)')) %>%
# rename(start_position=gene_start,
# end_position=gene_end) %>%
# filter(!is.na(entrezgene_id)) %>%
# filter(!is.na(hgnc_symbol))
```

Adding Genome Information to data set

```r
clearclipfinal <- hg18 %>% inner_join(clearclip, by = c(entrezgene_id = "gene.id",
    hgnc_symbol = "gene.symbol")) %>% distinct()
```

Converting human genome build

```r
# Obtaining chromosomal locations from miRNA:target
# interaction data set.

lift18 <- clearclipfinal %>% unite(start_end, c("start_position",
    "end_position"), sep = "-") %>% unite(location,
    c("chr", "start_end"), sep = ":") %>% dplyr::select(location)

# you can save current state of lift18 data frame
# write_tsv(lift18, 'data/lift18.txt')
```

Please note that we have provided the hg18 liftover data set under the data folder (lift18), in case of server errors/changes may occur. If you had problems generating lift18 data frame, please run the following code.

```r
lift18 <- read_tsv("data/lift18.txt")
```

```r
# In liftover site, Original Assembly was chosen as
# hg18 and New Assembly was chosen as hg38. After
# we uploaded 'lift18.txt' file to UCSC Liftover
# site, the output 'View Conversions' was
# downloaded as 'hg38clearclip.txt' (new locations
# of the genes on HG38), 'Display failure file' was
# download as 'lift18_del.txt' (deleted regions on
# the HG38 genome build)

deleted_lift18 <- read_tsv("data/lift18_del.txt", comment = "#",
    col_names = FALSE) %>% rename(Chromosome_loc = X1) %>%
    dplyr::filter(str_starts(Chromosome_loc, "chr")) %>%
    separate(Chromosome_loc, c("Chr", "End"), "-",
        remove = TRUE) %>% separate(Chr, c("Chr", "Start"),
    ":", remove = TRUE) %>% mutate(Start = as.numeric(Start),
    End = as.numeric(End))


# removing deleted location from CLEAR-CLiP data
# set

clearclipfinal <- clearclipfinal %>% dplyr::anti_join(deleted_lift18,
    by = c(chr = "Chr", start_position = "Start", end_position = "End"))

hg38clearclip <- read_tsv("data/hg38clearclip.txt",
    col_names = FALSE)

# adding new location information:
clearclipfinal <- clearclipfinal %>% dplyr::bind_cols(hg38clearclip) %>%
    rename(HG38build_loc = X1) %>% dplyr::mutate(Genom_build = "hg18") %>%
    dplyr::select(cluster = cluster.ID, chromosome = chr,
        start_position, end_position, strand = strand.y,
        hgnc_symbol, Ensembl_Gene_Id = ensembl_gene_id,
        Ensembl_Transcript_Id = ensembl_transcript_id,
        target_seq = target.map, miRNA, miR_seq = miR.map,
        seed_type = "seed match", Energy = MFE, HG38build_loc,
        Genom_build, region)
```

Seed type manipulation in CLEAR-CLiP data set

In CLEAR-CLiP data set, seed types were shown in detail. We adjusted as canonical and non-canonical.

```
clipdata_seed <- data_frame(seed_type = c("5mer_1",
    "5mer_2", "5mer_3", "6mer", "6mer.indel", "6mer.mm",
    "6mer_off.mm", "6merA1", "6merA1.indel", "6merA1.mm",
    "7merA1", "7merA1.indel", "7merA1.mm", "7merm8",
    "7merm8.indel", "7merm8,mm", "8mer", "8mer.indel",
    "8mer.mm", "NA"), seed_type_com = c("5-mer", "5-mer_noncanonical",
    "5-mer_noncanonical", "6-mer", "6-mer_noncanonical",
    "6-mer_noncanonical", "6-mer_noncanonical", "6-merA1",
    "6-merA1_noncanonical", "6-merA1_noncanonical",
    "7-merA1", "7-merA1_noncanonical", "7-merA1_noncanonical",
    "7-mer-8m", "7-mer-8m_noncanonical", "7-mer-8m_noncanonical",
    "8-mer", "8-mer_noncanonical", "8-mer_noncanonical",
    "none"))

clearclipfinal <- clearclipfinal %>% inner_join(clipdata_seed,
    by = "seed_type") %>% dplyr::select(1:11, seed_type = seed_type_com,
    Energy, HG38build_loc, Genom_build, region)
```

2.3 Integration of two experimental data set

CLASH and CLEAR-CLiP data were processed heavily and resulted in similar format after which they were concatenated.

```
experimentalmirnagene <- bind_rows(clashelwakfinal,
    clearclipfinal) %>% distinct()
```

2.4 Adding Coefficients of Interaction factors

Energy values in miRNA:target pairs were represented by high-throughput studies (Helwak et al. 2013; Moore et al. 2015) which were utilized in this study. On the other hand, we specified the other interaction factors (seed type and location of binding region on the target) as numeric values based on the previous studies.(Grimson et al. 2007) had compared the seed types' effect on target repression with few miRNA had canonical seed pairing in their study. Additionally, (Bartel 2009) and (Betel et al. 2010) have studied on functional and non-functional seed interactions. Based on results of these studies we compiled seed types of miRNA:target interactions as numeric values. We also have re-defined location of binding region on the target as numeric values, based on studies of (Hausser et al. 2013) and (Helwak et al. 2013). With this process, we handled this integrated data set in context of competitor behaviors and functionality of interactions.

In this step we added numeric interaction values as following

Firstly, we organized these values due to the fact that the regions were defined differently in two data set. After that, region effect was added as numeric values (shown in Table S3).

```
experimentalmirnagene <- experimentalmirnagene %>%
    mutate(region2 = str_replace_all(region, "NA",
        ""), region3 = str_replace_all(region2, "\\|",
        ""), region = str_replace_all(region3, c(`3'UTR` = "3UTR",
        `5'UTR` = "5UTR"))) %>% dplyr::select(-region2,
```

```
    -region3) %>% mutate(region_effect = case_when(region %in%
    c("3UTRCDS", "CDS3UTR", "5UTR3UTR", "CDS5UTR3UTR",
        "CDS3UTRintron") ~ "0.93", region %in% c("5UTRCDS",
    "CDS5UTR") ~ "0.42", region %in% c("CDS", "CDSintron") ~
    "0.42", region %in% c("3UTR", "3UTRintron") ~ "0.84",
    region %in% c("5UTR", "5UTRintron") ~ "0.01", region %in%
        c("intron", "") ~ "0.01", region %in% c("exon_unclassified",
        "") ~ "0.2", TRUE ~ NA_character_)) %>% mutate(region_effect = as.double(region_effect))
```

Secondly, we arranged seed type parameters, as in *Seed type manipulation* section, of CLEAR-CLiP data set to show as found in CLASH data set. The values added data set as numeric values (shown in Table S2).

```
seed_type_effect <- data_frame(seed_type = c("5-mer",
    "5-mer_noncanonical", "6-mer", "6-mer_noncanonical",
    "6-merA1", "6-merA1_noncanonical", "7-mer", "7-mer_noncanonical",
    "7-merA1", "7-merA1_noncanonical", "7-mer-8m",
    "7-mer-8m_noncanonical", "8-mer", "8-mer_noncanonical",
    "9-mer", "9-mer_noncanonical", "none"), seed_type_effect = c(0.05,
    0.04, 0.07, 0.05, 0.07, 0.05, 0.23, 0.19, 0.19,
    0.16, 0.25, 0.21, 0.43, 0.35, 0.43, 0.35, 0.01))
experimentalmirnagene <- experimentalmirnagene %>%
    inner_join(seed_type_effect, by = "seed_type")
```

The approaches to detect miRNA:target interactions are based a basic principle that is reading after isolation of miRNA:target chimerics. The data sets contain all readings from the chimeric miRNA:target pairs, isolated at a particular time. On the other hand, it could be said that the reading is performed in a snapshot fashion. Because of that, the methods can provide different chimeric interactions the same miRNA:target pair. We have preferred to select most effective interaction parameters for the same miRNA:target pairs that can exhibit various interactions. The step was performed as:

```
experimentalmirnagene <- experimentalmirnagene %>%
    dplyr::select(miRNA, Ensembl_Gene_Id, hgnc_symbol,
        Energy, seed_type_effect, region_effect) %>%
    distinct() %>% group_by(Ensembl_Gene_Id, miRNA) %>%
    mutate(seed_type_effect = ifelse(seed_type_effect ==
        max(seed_type_effect), seed_type_effect, max(seed_type_effect)),
        Energy = ifelse(Energy == min(Energy), Energy,
            min(Energy)), region_effect = ifelse(region_effect ==
            max(region_effect), region_effect, max(region_effect))) %>%
    ungroup() %>% distinct()

saveRDS(experimentalmirnagene, file = "data/experimentalmirnagene.RDS")
```

```
# the data frame generated up to now should match
# the data frame used in manuscript so the output
# should be 'zero' different rows.
dplyr::setdiff(experimentalmirnagene, readRDS("data/experimentalmirnagene.RDS"))
```

The context of data set was shown in Table S5 in Supplementary Tables.

3. Functions defined for ceRNA models and workflow of method

We defined the functions that can be used with R programming. Briefly, these functions process a given miRNA:gene data set and convert to graph object. All values that are significant in miRNA:target interactions are stored in edge variables and processed with formulations that were suggested in manuscript. The functions and steps of approach are explained as following (Figure S1) :

Conversion of data set: `priming_graph()` function processes the given data set that includes competing nodes in first variable and repressive miRNA nodes in second variable. If the affinity and/or degradation factors are specified in the function, factors are taken into account, are processed with defaults in vice versa. The formulations that are given in equations (1-4) are performed in this function. This step gives the graph object which contains *repression activity* values of miRNAs on paired target in steady-state.

Selecting trigger in graph: The data set is assumed as steady-state in previous step and the interaction parameters are calculated. In the network at steady-state conditions, the change is applied to the graph object for perturbation of steady-state. To provide the perturbation in the network the work-flow offer two methods: *update_variables* and *update_how*. The first, a new data set that is contained competing and miRNA names and current values of these are processed. The second option, the amount of the given node name in update_how function, is changed according to "how" argument.

Simulation of competing behavior of targets: After the change in the steady-state conditions, the network object tries to reach steady-state again. This process progresses as repeating of regulations after the spreading the changes in the network. In this step, simulation of regulations according to given cycle count in *simulate* function is applied. After each simulation cycle, the miRNA repression activities are re-calculated and the current values of competing elements are found and saved. The process is performed in the edge data and at the same time outputs of the calculations are carried from edge to node variables.

The nodes in the data set are handled as two type; miRNAs and competing (i.e., genes or targets). It is assumed in approach that while targets are degrading or inhibiting by miRNAs continuously, miRNAs reversibly used. If the trigger of the network is a miRNA, it maintains the current value of amount that provides by user. On the contrary, it tries to help this process to provide steady-state through the regulations on its amount, if a competing element is used as a trigger. The functions that are used in the this study are developed with R and are available in Bioconductor.

4. Perturbation analysis of Real/Real+ networks

To evaluate significant nodes through parallel processing in breast cancer patient network, perturbation on all nodes were triggered by 3-fold up-regulation with 10 iteration (cycle) due to small diameter of Real and Real+ networks. We analyzed perturbation efficiencies of all nodes in situation that accepts nodes with more than one percent change in expression as re-regulated (i.e, limit=1 argument in *find_node_perturbation()* function).

REFERENCES

Bartel, David P. 2009. "MicroRNAs: Target Recognition and Regulatory Functions." *Cell* 136 (2): 215–33. https://doi.org/10.1016/j.cell.2009.01.002.

Betel, Doron, Anjali Koppal, Phaedra Agius, Chris Sander, and Christina Leslie. 2010. "Comprehensive Modeling of microRNA Targets Predicts Functional Non-Conserved and Non-Canonical Sites." *Genome Biology* 11 (8): R90.

Grimson, Andrew, Kyle Kai-How Farh, Wendy K. Johnston, Philip Garrett-Engele, Lee P. Lim, and David P. Bartel. 2007. "MicroRNA Targeting Specificity in Mammals: Determinants Beyond Seed Pairing." *Molecular Cell* 27 (1): 91–105. https://doi.org/10.1016/j.molcel.2007.06.017.

Hausser, J., A. P. Syed, B. Bilen, and M. Zavolan. 2013. "Analysis of CDS-Located miRNA Target Sites Suggests That They Can Effectively Inhibit Translation." *Genome Research* 23 (4): 604–15. https://doi.org/10.1101/gr.139758.112.

Helwak, Aleksandra, Grzegorz Kudla, Tatiana Dudnakova, and David Tollervey. 2013. "Mapping the Human miRNA Interactome by CLASH Reveals Frequent Noncanonical Binding." *Cell* 153 (3): 654–65. https://doi.org/10.1016/j.cell.2013.03.043.

Moore, Michael J., Troels K. H. Scheel, Joseph M. Luna, Christopher Y. Park, John J. Fak, Eiko Nishiuchi, Charles M. Rice, and Robert B. Darnell. 2015. "miRNA-Target Chimeras Reveal miRNA 3'-End Pairing as a Major Determinant of Argonaute Target Specificity." *Nature Communications* 6 (November): 8864. https://doi.org/10.1038/ncomms9864.
